# Genetic Population Structure Accounts for Contemporary Ecogeographic Patterns in Tropic and Subtropic-Dwelling Humans

**DOI:** 10.1371/journal.pone.0122301

**Published:** 2015-03-27

**Authors:** Daniel J. Hruschka, Craig Hadley, Alexandra A. Brewis, Christopher M. Stojanowski

**Affiliations:** 1 School of Human Evolution and Social Change, Arizona State University, Tempe, Arizona, United States of America; 2 Department of Anthropology, Emory University, Atlanta, Georgia, United States of America; University of Bristol, UNITED KINGDOM

## Abstract

Contemporary human populations conform to ecogeographic predictions that animals will become more compact in cooler climates and less compact in warmer ones. However, it remains unclear to what extent this pattern reflects plastic responses to current environments or genetic differences among populations. Analyzing anthropometric surveys of 232,684 children and adults from across 80 ethnolinguistic groups in sub-Saharan Africa, Asia and the Americas, we confirm that body surface-to-volume correlates with contemporary temperature at magnitudes found in more latitudinally diverse samples (Adj. R^2^ = 0.14-0.28). However, far more variation in body surface-to-volume is attributable to genetic population structure (Adj. R^2^ = 0.50-0.74). Moreover, genetic population structure accounts for nearly all of the observed relationship between contemporary temperature and body surface-to-volume among children and adults. Indeed, after controlling for population structure, contemporary temperature accounts for no more than 4% of the variance in body form in these groups. This effect of genetic affinity on body form is also independent of other ecological variables, such as dominant mode of subsistence and household wealth per capita. These findings suggest that the observed fit of human body surface-to-volume with current climate in this sample reflects relatively large effects of existing genetic population structure of contemporary humans compared to plastic response to current environments.

## Introduction

Bergmann’s and Allen's rules are perhaps the best-known of all ecogeographical principles. They propose that animal body forms adapt to local climates so that cold-adapted populations tend to have more compact bodies and shorter limbs that reduce the ratio of surface area to heat-producing mass or volume. Conversely, heat-adapted populations tend to have less compact frames and longer limbs that improve the capacity for dissipating heat. While differing in focus—on body size and limb proportions, respectively—both rules hinge on a deeper ecogeographic prediction that an organism's surface area to volume will increase in warmer climates and decrease in cooler climates. Numerous studies have shown this general pattern to hold in humans, with populations living in the tropics having less compact frames and those in colder climates having more compact builds [1–12]. This is also consistent with experimental evidence that taller and thinner human bodies are better at dissipating heat [13, 14].

Though ecogeographic predictions have been confirmed many times in humans, the proximate pathways driving the implied adaptive fit in humans and other animals are poorly understood [1, 13–15]. Contemporary variation in body form can arise from genetic differences across human populations that have arisen through a variety of processes, including natural selection (either stabilizing or directional), gene flow, genetic drift, and mutation. For example, if there has been a long-term correlation between a population's past and current climates and natural selection has also favored genetic adaptations for body form that regulate heat loss and conservation, then we would expect to see some fit between contemporary climate and body form. It is also possible that an observed fit could arise due to patterns of migration and gene flow that are correlated with geography and climate [16, 17].

Contemporary variation in body form can also arise from plastic responses to current environments. For example, moderate and chronic undernutrition—more common in warmer tropical areas—can produce thinner adult bodies [13, 18–20]. Human bodies are also proposed to respond over the course of development to thermic stress in a way that fits the local environment [1, 14]. These arguments are consistent with findings from other animals. For example, experimentally increasing ambient temperature leads to increased long bone growth in mice [21]. Similarly, a longitudinal study of body mass among red-billed gulls showed that the mean population body size declined over 47 years of increasing temperatures, but there was no evidence that this was due to directional genetic selection, suggesting a plastic response to climate change [22]. Recent evidence from human populations constrains the timing of these plastic responses by showing that population variation in body build among humans emerges in infancy and early childhood. This suggests that observed climate-related differences in human body form likely arise in utero or very early in development [10, 11].

To the degree that plastic responses to the current environments are responsible for some of the observed fit between climate and human body form, we would expect that two groups with close genetic affinity but living in different climatic conditions should exhibit different body forms that reflect these differing climatic conditions. Specifically, the body shapes of people living in a warmer climate should exhibit greater average surface area to volume than the average body shapes of genetically similar people living in a cooler climate. On the other hand, if most of the differences in body form are due to genetic differences, then we would expect: (1) that when comparing groups with similar genotypes, there should be little or no additional effect of contemporary climate on body form, and (2) that groups with similar genotypes should have more similar body forms.

Our goal here is to assess the degree to which the contemporary fit between human body form and climate can be attributed to plastic responses to current conditions independent of the genetic affinity among populations. To do this, we integrate anthropometric, genetic, and climatic data from 80 ethnolinguistic populations in Africa, Asia, and the Americas. To assess the relative compactness of human bodies, we use standard measures of weight relative to height in humans—body mass index (BMI) for adults and weight-for-height for children—that have been used in prior studies of ecogeographical rules in humans [1–3, 10, 11]. Widespread increases in adiposity are a very recent but globalizing phenomenon that mask underlying variation in body form, making unadjusted body form a noted limitation of prior studies of ecogeographical rules [2, 23]. To deal with this issue, we apply the novel concepts of basal body mass index for adults and basal weight-for-height in children as comparative population measures of body mass relative to height that largely removes the effects of recent nutrition transitions [11, 24]. Prior research has shown that there is a lower limit to the mean BMI of populations surviving with extremely scarce resources [24], and basal body mass index (or basal weight-for-height) is defined as the mean body mass index (or weight-for-height) of populations without sufficient resources to accrue additional mass. These estimates of basal body mass have been shown to be independent of country-level wealth and fat-linked disease prevalence [24].

For a measure of genetic affinity between populations, we use estimated proportion of ancestry from 14 genetic clusters identified by Tishkoff et al. [25]. A number of models of human genetic population structure have been proposed [26, 27]. We use Tishkoff's 14 cluster model for two reasons: (1) it contains publicly available estimates that can be linked to a large number of ethnic groups in existing Demographic and Health Surveys, and (2) the estimates are sufficient to account for a very large portion of variation in human body form as will be shown later.

For measures of heat and cold stress, we use three measures of mean, minimum, and maximum local temperature from ecological databases. The systematic anthropometric data from Demographic and Health Surveys is a strength of the study, but it also limits the sample to tropical- and subtropical-dwelling humans and thus excludes populations living in colder temperate regions. This restricted sample still shows ecogeographical associations of similar magnitudes to those observed in studies including populations from colder climates. However, given that two different adaptive processes may influence body build—adaptations to heat stress and adaptations to cold stress—the current findings are limited to theories based on adaptations to heat stress.

In this paper, we combine these estimates of genetic affinity, basal BMI and weight-for-height, and local climate, as well as environmental variables from 80 contemporary populations in Africa, southern Asia, and the Americas, to assess if current fit between climate and body form in tropical and subtropical-dwelling human groups is best explained as plastic responses to current conditions, a reflection of existing genetic diversity, or combinations of these.

## Materials and Methods

### Sample inclusions and data sources

Data on height and weight, household wealth, age, education and rural-urban residence were available for populations from nationally representative, repeated cross-sectional household Demographic and Health Surveys (DHS) datasets in 64 countries, standardized to permit cross-country comparisons [28] (available at measuredhs.com). DHS surveys conducted between 1991 and 2011 were included. To minimize wealth effects, we also restricted our analysis to individual households with estimated wealth less than 4000 USD per capita (2011 international units, purchasing power parity). For each country, there were between one and five surveys conducted in different years. Detailed protocols for DHS surveys as well as survey data are available at measuredhs.com.

For genetic population structure, we use estimates from Tishkoff et al. [25]. We analyzed 80 unique populations reported by Tishkoff et al. with comparable populations measured by the DHS. In addition to 18 populations from South Asia and 58 populations from Africa, genetics and anthropometric data exist for two populations from the Americas (Guatemala and Colombia) and two from southeast Asia (Cambodia and Timor L'Este) [25]. Although there are other models of human population structure [26, 27], the Tishkoff et al. paper provides readily available estimates of genetic affinity. We will also show that despite potential errors introduced by this model, the estimates still provide very good fit to morphological data.

For estimates of basal BMI in women, we excluded pregnant women as well as women with more than primary education, and include a dummy variable controlling for urban residence [24]. To control for lactation, we also include a dummy variable for whether women were currently breastfeeding on the day of the interview. For men, we excluded individuals with more than primary education and include a dummy variable controlling for urban residence. For children, we included most recently born living children (ages 0 to 59 months) of women ages 20–49 who have no more than primary education [11]. We also include a control for urban residence.

The anthropometric (Demographic and Health Surveys), economic (World Bank Indicators), genetic (supplementary materials in Tishkoff et al.), and climatic data (WorldClim) are all publicly available. The study was approved by Arizona State University's Office of Research Integrity and Assurance IRB (Protocol #1302008836).

### Variables

#### Body Mass Index

In each of the country samples, height and weight measures were taken by trained technicians. BMI was calculated as weight (kg)/height (m)^2^ and weight-for-height was calculated as weight (kg)/height (m). We analyzed data on BMI for young adults (20–34 years) and weight divided by height for children (0–59 months). These measures are highly correlated (R^2^ > 0.90) with body surface-area-to-volume estimates in both adult [29] and child [30] samples.

#### Household wealth per capita

We use estimates of household wealth that integrate information about: (1) relative household wealth in a country in each survey year, (2) the wealth Gini coefficient approximating the percentage of total country wealth owned by each household based on its relative rank in wealth [31], and (3) country-level wealth in the survey year approximated from country-level gross domestic product [24, 32]. This procedure provided a measure of household wealth in terms of internationally and inter-temporally comparable units—purchasing power parity in constant 2011 international units [24]. We use the logarithm of this wealth measure as the key predictor for partialling out effects of wealth, as body mass has been shown to scale logarithmically with wealth in prior analyses [24].

#### Ethnolinguistic populations

Ethnolinguistic populations were defined by respondent's ethnicity or language as recorded in the Demographic and Health Survey. We focus on those ethnolinguistic populations in Africa, southern Asia and the Americas analyzed by Tishkoff et al. (2009) where the DHS surveys include relevant data (S1 Fig. and S1 Table). We matched groups from Tishkoff et al. with ethnolinguistic designations in the DHS surveys based on common ethnic identification and native language (S1 Table). For Hausa, Fulani, and Maasai groups that show considerable geographic spread, we matched those DHS data by country and region to three Fulani groups, two Hausa groups, and two Maasai groups in Tishkoff et al.'s sample (S1 Table).

#### Genetic affinity

Using 121 African and 60 non-African ethnolinguistic populations, Tishkoff et al. inferred 14 ancestral population clusters using 1327 nuclear microsatellite and insertion/deletion markers. Tishkoff et al. labeled these clusters in terms of related regional and linguistic groupings [25]. Nine of the clusters were associated with groups in sub-saharan Africa (labeled Fulani, Nilo-Saharan, Chadic, S. African Khoesan, Niger-Kordofan, Cushitic, Hadza, Sandawe, W. Pygmy), three were from Eurasia (East Asia, India, European), one was associated with Oceania (Oceania) and one with the Americas (Native American). For each modern ethnolinguistic population, Tishkoff et al. also estimated the proportion of genetic ancestry from each of these 14 inferred ancestral clusters. These 14 clusters do not necessarily reflect bounded historical populations, but they do provide a straightforward tool for estimating the genetic affinity of current ethnolinguistic groups. Specifically, two ethnolinguistic groups that have on average more similar proportion of estimated ancestry from these 14 clusters are also more likely to have closer genetic affinity. Here, we focus on the proportion of genetic ancestry from the thirteen clusters for which at least one of the ethnolinguistic groups available in the DHS datasets had at least 10% inferred ancestry from that cluster. These thirteen clusters include Fulani, Nilo-Saharan, Chadic, S. African Khoesan, Niger-Kordofan, Cushitic, W. Pygmy, Sandawe, Oceania, East Asia, India, European, and Native American. This excludes the Hadza subcluster.

#### Latitude and longitude

The latitude and longitude for each ethnolinguistic unit was identified as the value at the sampling longitude and latitude from Tishkoff et al. [25].

#### Local climate

The following climatic variables were extracted from the WorldClim global climate database of average values between 1950–2000—mean annual temperature, minimum temperature of coldest month, and maximum temperature of warmest month [33]. The value for each ethnolinguistic unit was identified as the value at the sampling longitude and latitude from Tishkoff et al. [25].

#### Operationalizing basal BMI and basal weight-for-height

We operationalize population *basal BMI* as the expected body mass index of an adult with insufficient resources to accrue excess body mass. Body mass index is often used as a measure of obesity or excess body fat. However, human populations can also differ substantially in the quantity of fat free mass per unit height. Thus, at low levels of body fat, body mass index is also a measure of lean compactness or stockiness. Studies that have measured fat and lean mass using x-ray methods in select populations have demonstrated that population variation in compactness is primarily variation in lean mass [34, 35].

There are several environmental factors that can lead to increasing fat mass and thus bias BMI (or weight-for-height among children) as a measure of underlying body compactness [2, 5]. To assess a population's *basal BMI*, we estimate the BMI of a young adult (ages 20–34) removing the effects of key contemporary environmental variables known to influence BMI, including household wealth, urban residence, and educational attainment [24, 36]. When applied to populations in low and middle income countries, this method produces reliable estimates of population differences consistent with direct measures of lean body composition, which is independent from country-level wealth and the prevalence of fat-linked disease [24].

As in prior validated work, we estimate population *basal BMI* of an ethnolinguistic group as the expected BMI of young adults with little education (primary education or less) living in rural households with very little household wealth (cash equivalent of 300 USD per capita in constant 2011 PPP international units). We focus on young adults (ages 20–34 years) who have presumably reached full height, but have not yet been able to store much excess fat [24]. Among women, we exclude pregnant individuals and adjust for current lactation. Basal BMI is formally estimated with a mixed model predicting BMI with the following fixed effects: log(wealth), age in years, breastfeeding status, rural residence, and an interaction between log(wealth) and age. We include ethnolinguistic group as a random effect. Our estimate of basal BMI for an ethnolinguistic group is the empirical best linear unbiased predictor (EBLUP) for that group [24]. Our approach here is identical to that described in earlier work, except for three modifications [24]. First, it uses improved wealth measures that are in 2011 PPP international units [32]. Second, it includes 20-34-year-olds as well as urban populations to ensure sufficiently large samples for each ethnic group. Finally, it uses ethnolinguistic unit rather than country as a random effect to capture between-group variation in basal body mass index.

We use a similar approach to estimate basal weight for height (bWH) among children (0–60 months) [11]. Basal weight-for-height is formally estimated with a mixed model predicting weight-for-height with the following fixed effects: log(wealth), age in months, rural residence, and an interaction between log(wealth) and age. Due to non-linear relationships between age and WH in children, we include age in months as a categorical variable with 24 months as the reference category. We include ethnolinguistic group as a random effect and use the empirical best linear unbiased predictor (EBLUP) for each ethnolinguistic group as the estimate of basal WH [24].

### Analysis: Assessing effect of climate on body form after controlling for genetic similarity

We assessed the effects of climatic variation and genetic affinity on population variation in bBMI and bWH across ethnolinguistic groups in each of four demographic categories (male children, female children, male adults, female adults). For the crude effect of climatic variation we regressed population bBMI and bWH on the three climatic variables using OLS regression. Due to collinearity between these three climatic variables, we considered each climatic variable in an independent regression.

To assess the potential effect of genetic affinity on bBMI and bWH, we fit OLS regressions predicting bBMI and bWH from the proportion of an ethnolinguistic group's genetic ancestry derived from each of 13 genetic clusters identified by Tishkoff et al.—Fulani, Nilo-Saharan, Chadic, S. African Khoesan, Sandawe, Niger-Kordofan, Cushitic, West Pygmy, East Asia, India, Oceania, and Native American. For example, Kikuyu were estimated by Tishkoff et al. to have 43% ancestry from the Niger-Kordofan cluster, 36% from Cushitic, 8% from Nilo-Saharan, 6% from Sandawe, and 1% or less from each of the other clusters. We fit a regression including each of these cluster percentages as an independent variable. Since the percentage of genetic ancestry from these 13 genetic clusters adds to 100% in each ethnolinguistic group, we regressed bBMI and bWH on percentage of ancestry from twelve of the genetic clusters (e.g., 12 variables), excluding the proportion of Niger-Kordofan ancestry for use as a reference category. We use Niger-Kordofan ancestry as the reference category given that it represents the component with the largest share of ancestry in this sample of ethnolinguistic groups.

The regression model for bBMI without other covariates would be:

$${bBMI}_{j}\;=\;\beta_{0}+\sum_{i\;=\;1}^{12}\beta_{i}X_{ij}+\varepsilon_{j}$$

Here *β*_*i*_ is the regression coefficient for cluster i, and *X*_*ij*_ is the proportion of ancestry of ethnolinguistic group j from cluster i. Based on this, the regression intercept *β*_0_ is the bBMI estimate for an idealized individual of 100% ancestry from the Niger-Kordofan component. By similar logic, the parameter estimate for Fulani genetic ancestry is the degree to which a hypothetical individual of 100% ancestry from the Fulani cluster diverges from the reference Niger-Kordofan value.

For each ethnolinguistic group, we refer to the predicted values of bBMI and bWH from this regression model as genetic affinity-predicted bBMI and affinity-predicted bWH. We use these genetic affinity-predicted values for mediation analyses (described later) examining how body form predicted from genetic affinity accounts for the observed relationship between contemporary temperature and current body compactness.

As stated earlier, if ecogeographical associations arise in part from plastic responses to contemporary climate, then we expect that genetically similar groups living in different contemporary climates should show body form differences consistent with ecogeographical rules. On the other hand, if ecogeographical associations largely reflect genetic population structure, arising from past processes of selection, drift, or gene flow, then we should see little difference in body forms between genetically similar groups that are living in different contemporary climates. To assess these hypotheses, we compare the following models, (1) temperature alone, (2) genetic affinity alone, (3) temperature and genetic affinity together. There were 12 temperature alone models (body mass outcome for each of 4 age/gender groups x 3 temperature variables). There were 4 models for genetic affinity alone (one for the body mass outcome for each of 4 age/gender groups). Just as there were 12 temperature alone models, there were also 12 temperature and genetic affinity models. We use two assessments of fit, both of which penalize for adding parameters to the model—Adjusted R^2^ and the Akaike Information Criteria (AIC).

To assess how much of the effect of climatic variables on body form is accounted for by affinity-predicted body mass, we use a bootstrap mediation analysis. While model comparison using AIC allows us to select models with minimal complexity that best fit the data, mediation analysis gives information about how the addition of one variable to a model reduces the effect of another variable. In this case, the mediation analysis identifies how much of the raw effect of temperature on body form is reduced when including genetic affinity-predicted body form as a covariate [37]. In this way, it gives the direct effect of climatic variables as well as the portion of this effect accounted for by genetic affinity [37]. Due to the small number of cases (n = 20) for male adults relative to the number of genetic ancestry variables (k = 12), we use the affinity-predicted value of bBMI from females in the mediation analyses for adult males. All analyses were conducted in SPSS 22.0 [38].

One concern when comparing populations with varying degrees of genetic relatedness is the problem of non-independent observations. Given that a phylogenetic tree does not exist for these populations, it is not feasible to use phylogenetic methods to adjust inferences [39]. Instead, we used the variables capturing genetic affinity among groups—Tishkoff's cluster membership variables—to assess to what degree the effect of climatic variables can be accounted for by genetic affinity among groups.

A number of processes, including selection and neutral processes such as gene flow, genetic drift, and mutation, could lead to genetic variation in body form [16]. Selection for adaptive body forms may create the current fit with ecogeographic predictions. However, a fit between body form and climate might also arise from neutral processes of migration that are correlated with climate and geography. We assess the degree to which current variation may have arisen from neutral processes as follows. The expansion of our species out of the African continent has created a global pattern of neutral variation in both genetic variants and morphological traits [16, 17]. To assess the degree to which the observed variation in body form can be attributed to this expansion out of Africa, we use the approach laid out by Betti et al. that examines the degree to which within-population variation in body form declines with distance from Central Africa [16]. We do this by including the distance between an ethnolinguistic group's current location and Central Africa in an OLS regression predicting within-population variance in basal body mass (bBMI and bWH). Following Betti et al., we calculate the Haversine distance between Central Africa (8S, 25E) and the location of each ethnic group, using Sinai Peninsula (30.07N, 33.7E) as the waypoint out of Africa, Bering Strait (65.78N, 169.97W) as the waypoint to the Americas, Panama (13.5N, 86.2W) as the route to South America, and Thailand (16.13N, 98.35E) as the route to Oceania.

## Results

### Sample characteristics

There were 80 ethnolinguistic groups identified in Demographic and Health Survey datasets available from MEASURE DHS with information on genetic ancestry from Tishkoff et al. [25]. A total of 52886 female and 55605 male children (0 to 60 months) and 107296 women (20 to 34 years) from these 80 linguistic groups were analyzed. Data were limited to 20 ethnolinguistic groups for men (n = 16897, 20 to 34 years). Samples ranged from 3 to 8898 in female children, 1 to 9782 in male children, 6 to 19812 in women and 2 to 5587 in men (S1 Table).

### Associations across gender and age group

Basal WH estimates were highly correlated between boys and girls (R^2^ = 0.86, n = 80, p < 0.001). Across the 20 ethnic groups with data for men and women, estimates of bBMI were also correlated across sexes (R^2^ = 0.80, n = 20, p < 0.01). The correlation between child bWH and adult bBMI was high for females (R^2^ = 0.70, n = 80, p < 0.001) and moderate for males (R^2^ = 0.59, n = 20, p < 0.01). The moderate to strong relationship between adult bBMI and child bWH, especially among females, confirm earlier findings that a large portion of cross-population differences in body form arise very early in development [11].

### Regional variation in bBMI

The average adult bBMI varied substantially across major world regions and was consistent with prior estimates of bBMI conducted at the country level [24] (Table 1). These regional estimates are also highly correlated between boys and girls, and between women and children, although the sample size is small (R^2^ = 0.80–0.99, n = 4).

**Table 1:** Average adult basal BMI (kg/m^2^) and child basal WH (kg/m) by major world region.

	****Adults****		****Children****	
	****Female bBMI****	****Male bBMI****	****Female bWH****	****Male bWH****
**South Asia**	19.5 (1.8, 18)	19.0 (0.5, 14)	11.1 (0.4,18)	11.7 (0.4,18)
**Southeast Asia**	20.0 (0.4, 2)		11.9 (0.0,2)	12.3 (0.0,2)
**Sub-Saharan Africa**	21.3 (1.0, 58)	20.2 (1.2, 6)	12.5 (0.4,58)	12.9 (0.4,58)
**Americas**	23.7 (0.9,2)		12.8 (0.2,2)	13.2 (0.2,2)

Numbers in parentheses are standard deviation and number of ethnolinguistic groups.

### Ethnolinguistic variation in bBMI

In addition to these reliable macro-regional differences, there is considerable variation across the 80 ethnolinguistic groups within both Africa and South Asia. The Nuer, a population commonly used to exemplify slender body builds in anthropological textbooks, had the lowest estimated value for women (bBMI = 18.4) and men (bBMI = 18.5), and one of the lowest for children (average bWH = 12.1) in the African sample. These values are 4.8–5.2 kg/m^2^ lower than female bBMI and 1.3–1.5 kg/m lower than the average child bWH found in the Bamoun and Wimbum groups in central Cameroon, which have the highest values in the African sample. The differences across South Asian samples were also substantial. Groups from northern India, such as Punjab, Kashmiri, Pathan, and Balochi, had body masses much greater than populations from other parts of South Asia (bBMI = 19.7 to 23.4 for women, bBMI = 19.4 to 20.0 for men, average bWH = 12.0 to 12.6 kg/m). The South Asian group with the highest basal body mass (Brahui female bBMI = 23.4 and average bWH = 12.6) was 5.5 kg/m^2^ higher in adults and 1.1 kg/m higher in children than Gujarat populations from midwestern India (Gujarat female bBMI = 17.9 and average bWH = 11.5), which had some of the lowest values in the South Asian populations.

### Associations with temperature

**Fig 1:**
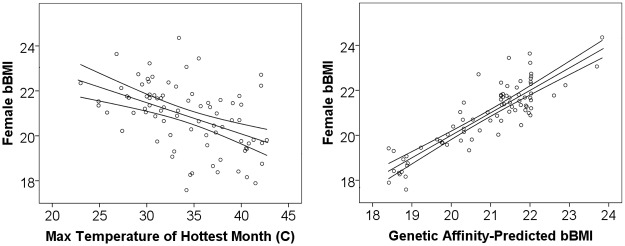
Relationship between basal Body Mass Index among adult females and (a) maximum temperature of hottest month, (b) genetic affinity-predicted values.

Both maximum temperature in the hottest month and mean annual temperature were moderately correlated with bBMI in adults and bWH in children (Adj. R^2^ = 0.14–0.28, Table 2). There was no relationship between body build and minimum temperature of the coolest month (Adj. R^2^ < 0.01 for all four samples, p > 0.10). The lack of association with cold stress in this sample may result from the sample's restriction to tropical and subtropical populations. However, it is also consistent with past results from samples drawn from a much wider range of latitudes [40]. Fig. 1a shows the relationship between maximum temperature of the hottest month and female bBMI among 80 ethnolinguistic groups (see S2 Table for the same results for child bWH).

**Table 2:** Coefficient of determination (Adjusted R^2^) of models predicting basal body mass based on climatic variables and genetic affinity (n = 80 for all except adult male populations, n = 20).

	Adults		Children	
Model	Female bBMI	Male bBMI	Female bWH	Male bWH
**1. Maximum temp.**	0.21	0.22	0.28	0.25
**2. Mean temp.**	0.14	0.21	0.18	0.14
**3. Minimum temp.**	0.00	0.00	0.00	0.00
**4. Genetic Affinity**	0.74	0.60^a^	0.58	0.50
**5. Affinity+Max temp.**	0.77	0.62^a^	0.62	0.54

^a^ Model based on affinity-predicted bBMI from full adult female sample due to small sample size in adult males.

All effects statistically significant at alpha = 0.05 level, except for associations with minimum temperature

### Controlling for Genetic Affinity

When we regress female bBMI on the twelve variables indicating percentage of ancestry from the twelve inferred genetic clusters identified by Tishkoff et al. [25], we achieve a much better prediction of bBMI than the temperature-based model (Adj. R^2^ = 0.74, n = 80, p < 0.001) with a substantial improvement in model fit (AICc = 155.2 compared to 275.2 for best-fitting model based on maximum temperature). This is also true for the other two large child samples (male child Adj. R^2^ = 0.50, female child Adj. R^2^ = 0.58, n = 80, p < 0.001) with comparable improvements in model fit (AICc = 48.1 and 58.5 compared to 106.6 and 109.1 for best-fitting model based on maximum temperature). Because the adult male sample was not sufficiently large to run the full regression with 12 parameters, we predicted adult male bBMI from affinity-predicted bBMI from the full adult female sample. Again we find substantial model improvement over the temperature-based model (AICc = 40.9 compared to 55.5 for the best-fitting model based on maximum temperature). Adding temperature to the genetic affinity model did slightly improve fit among women and girls (AICc = 149.9 vs. 155.2, 46.3 vs. 48.1) but not boys or men (AICc = 59.1 vs. 58.5, 40.9 vs. 40.9).

The affinity-predicted values of bBMI and bWH for each ethnolinguistic group are strongly correlated across the three full samples—adult females, female children, and male children (R^2^ = 0.88 to 0.97, n = 80, p < 0.001). These affinity-predicted values of adult females and children also strongly correlate with bBMI values in the smaller male sample (Adj. R^2^ = 0.54 to 0.61, n = 20, p < 0.001). Taken together, these findings suggest these affinity-predicted values of basal body mass capture similar variation in child and adult samples of both sexes across ethnolinguistic groups. Fig. 1b illustrates the high degree to which bBMI modelled by genetic affinity approximates measured bBMI (see S2 Fig. for similar results for child bWH). The clustering of basal BMI values around 22 are groups with high levels of Niger-Kordofan ancestry that are not differentiated on genetic affinity.

**Fig 2:**
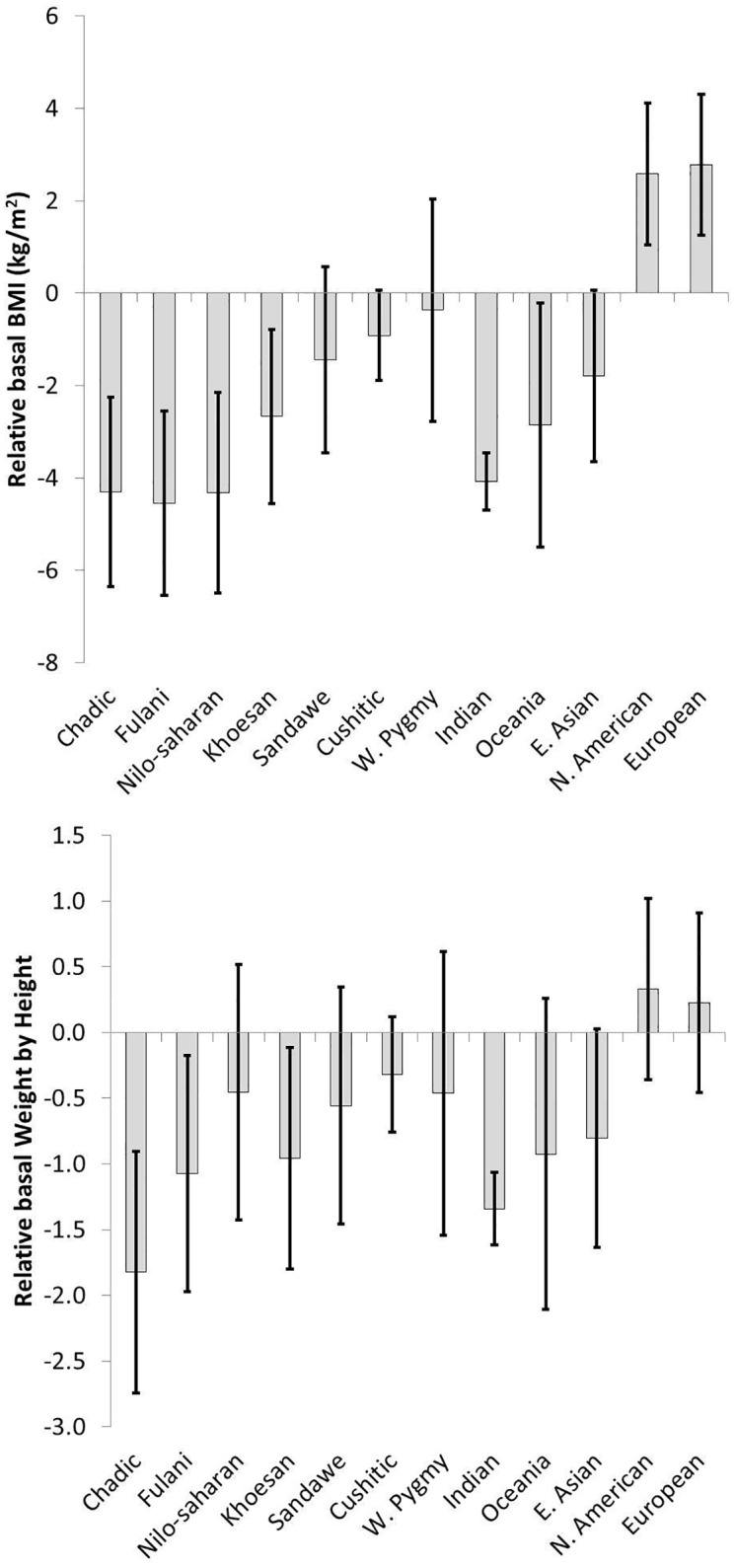
Estimated adult basal BMI and child basal WH for an individual of 100% ancestry from each of Tishkoff's genetic components. Niger-Kordofan is the reference category. Bars are 95% confidence intervals.

In addition to providing affinity-predicted estimates of bBMI for each of the 80 ethnolinguistic units, the genetic affinity model gives us some insight into how bBMI and bWH are associated with the 13 genetic clusters inferred by Tishkoff et al. [25]. Specifically, the regression intercept and 12 regression slopes provide estimates of bBMI and bWH for each of the 13 ancestral clusters inferred by Tishkoff et al [25]. The regression intercept from the model predicting female bBMI provides the expected bBMI for a woman of hypothetical 100% ancestry from the reference cluster of Niger-Kordofan (22.2 (95% CI = 21.8,22.5)). The remaining twelve regression coefficients provide the expected basal BMI (or WH) of a hypothetical individual with 100% ancestry from each of the remaining twelve genetic clusters. Fig. 2a depicts the bBMI deviation of each of the twelve remaining genetic clusters from Niger-Kordofan. Fig. 2b shows the comparable values for children's bWH. These coefficients show considerable and consistent variation across the genetic clusters in both the female adult sample and the child samples. Among predominantly African clusters, there is a substantial reduction in bBMI and bWH for the Chadic and Fulani genetic clusters (between -4.3 to -4.5 kg/m^2^ and -1.0 to -1.9 kg/m) and less so for Khoesan, Sandawe, Cushitic and West Pygmy components (-0.4 to -2.7 kg/ m^2^ and -0.2 to -1.0 kg/m). The one notable discrepancy is Nilo-Saharan which has substantial reductions in bBMI for adults (-4.3 kg/m^2^), but not for children (-0.4 kg/m). Among Asian and Pacific-associated components, Indian and Oceanic have values comparable with Chadic, Fulani and Nilo-Saharan, while East Asian values are closer to Cushitic, Sandawe and W. Pygmy. The Native American component is the one component that is statistically larger than the Niger-Kordofan reference in adults, consistent with past findings of higher bBMI among American populations [24].

When accounting for the effect of affinity-predicted body build, the direct effect of contemporary climate on contemporary bBMI or bWH is small (Additional Adjusted R^2^ explained by temperature variables is 0%-4%). Moreover, the AICc increases among males and decreases only slightly among females when including temperature in the genetic affinity model. This suggests that temperature variables do not substantially improve model fit over and above genetic affinity. Formal mediation analyses show that affinity-predicted bBMI mediates most of the effect of maximum temperature on adult bBMI (73% of the effect for women and 44% of the effect for men) and child bWH (70% of effect among both boys and girls). Bootstrap tests of mediation indicate that all of the indirect effects of genetic affinity are highly significant (p < 0.01 in all cases). Conversely, the direct effect of contemporary temperature on body form was not statistically significant among boys or men and was significant, though weak, among girls and women (p < 0.05) [37]. These results are similar when considering mean temperature. Given the low correlations between body build and minimum temperature, we did not conduct the mediation analysis for that climatic variable.

### Alternative hypotheses

It is possible that the association with genetic affinity is accounted for by correlated ecological variables such as diet or economic resources. However, across the 80 ethnolinguistic groups, neither dominant subsistence strategy nor mean household wealth significantly predicts basal BMI or WH (Adj. R^2^ = 0.01) for any of the three full samples in women or children of either sex. Adding subsistence type or wealth to the model based on genetic ancestry does not change estimates or inferences of the effect of genetic subcluster membership on bBMI. Indeed, adding these variables uniformly worsens model fit (e.g. AICc goes up). Another possibility is that these associations are due exclusively to macro-regional variation. However, the genetic ancestry model also predicts substantial variation *within* continents (n = 20 for southern Asia including Cambodia and East Timor, n = 58 for sub-Saharan Africa) for—adult females (Adj. R^2^ = 0.71 in southern Asia, Adj. R^2^ = 0.59 in sub-Saharan Africa, p < 0.01), female children (Adj. R^2^ = 0.43 and 0.32 respectively, p < 0.01) and male children (Adj. R^2^ = 0.21 and 0.32 respectively, p < 0.01). As a macro-level check on the possibility that other ecological variables (drought frequency, suitability of soils for especially nutritious crops, or incidence of disease) may confound the relationship between genetic affinity and body form, we included latitude and longitude of ethnolinguistic groups in a regression with the genetic affinity variables. Neither the coefficients nor the inferences for the genetic affinity variables changed substantially with the inclusion of these variables (S2 Table).

### Assessing divergence from neutral models

Among women and children, there was no significant correlation between the distance from Central Africa and within-group variance in bBMI or bWH. Among men, within-group variance in bBMI actually slightly *increased* (p = 0.05) with increasing distance from Central Africa. These findings do not support the argument that variation in basal body mass is due to a serial founder effect arising from migration out of Africa.

## Discussion

Consistent with the ecogeographic predictions relating climate and body form, basal body build is associated with local climatic variables in both children and adults in these tropic and subtropic samples at magnitudes comparable with studies conducted with more latitudinally diverse populations (21–22% of variation in basal BMI and 25–28% in basal WH). However, nearly all of this heat-related variation in bBMI and bWH is accounted for by genetic affinity, and basal body build predicted by genetic affinity also explains far more additional variation beyond local temperature—60 to 74% of total adult variation and 50 to 58% of total child variation in basal body form. Finally, adding temperature to the genetic affinity model does not improve model fit. These results suggest that a large part of the ecogeographic associations in this sample can be attributed to genetic population structure, perhaps reflecting longer-term genetic adaptations to climatic stressors or gene flow and genetic drift that are correlated with geography and climate. Plastic responses to climatic stressors or current climate-related food availability may still account for some of the conformity of human populations to ecogeographic predictions by both shifting population means and constraining variation. However, the current findings place some limits on the degree to which plastic responses alone are responsible for this variation.

Our analyses confirm striking variation in body build across contemporary human populations in Africa, Asia, and the Americas. Within sub-Saharan Africa alone, there was a 5.2 kg/m^2^ difference between the adult populations with the thinnest (i.e., Nuer of Ethiopia) and stockiest (i.e., Bamoun and Wimbum of Cameroon) body forms. This variation is consistent across sexes, and given the moderate to strong correlations between body build of young children and adults, much of this variation likely arises very early in development [10, 41]. The wide range of variation in contemporary body form is also consistent with the variation in body form observed among geographically dispersed *Homo sapiens* remains dated from the last 100K years [42].

In models predicting body form from genetic affinity, adult populations of predominantly Fulani, Nilo-Saharan, Chadic, and South Asian ancestry are expected to have the least compact body types, and populations with Native American and European ancestry are expected to have relatively compact body types. Relatively recent population migrations also illustrate how population movements can lead to substantial divergences between climate and body form in short time periods, a controversial issue in the anthropological literature on human variation [43]. For example, ethnolinguistic groups that have high affinity with Native American (Colombian and Guatemalan) and European components (Brahui, Mozabite, Pathan) have much higher female bBMI than expected (+2.2 to 3.4 kg/m^2^). This is consistent with findings from other investigators that Native American body forms, even those from tropical areas, appear to be derived from much colder ancestral Siberian environments [44] [6, 7, 45]. Conversely, Nilo-Saharan-descended Maasai living in Tanzania and Kenya have far less compact body builds than expected given the relatively cooler climate in these regions than ancestral Nilo-Saharan regions (1.6 kg/m^2^ lower BMI than expected, Fig. 1a). These examples suggest that divergences from ecogeographical expectations may not require arguments about the adaptive fit with the current environment, as has been proposed for stockier body forms among Polynesians [5]. Rather, such divergences may simply reflect selection for compactness in the deep past coupled with migrations into different climates or random processes of drift and gene flow. It is also possible that cultural tools that have permitted humans to regulate their temperature (e.g. constructed shelter, clothing) have attenuated the former processes of selection and permitted a greater impact of random genetic processes on body form.

The clear trend towards lower bBMI or bWH among those living in areas with the highest maximum and mean temperatures is consistent with the idea that selection over the long term leads to body shapes that maximizes surface area relative to body mass to facilitate heat loss. Here, we observe no association between bBMI or bWH and climate during the coldest months. This is consistent with a stronger effect of maximum (rather than minimum) temperature in human post-cranial remains over longer time scales [46]. This may also be an artifact of our sample not including many populations living in cold areas. In line with most current research, we have focused on fit of body form with one specific environmental variable—local temperature. However, there are other environmental variables that may also influence body form. In environments of greater pathogen stress, bodies may shunt resources away from muscle mass to improve immune function [47]. Some evidence also suggests that shorter limb length may confer advantages in more rugged terrains—the so-called mobility hypothesis for limb length—influencing overall body build [48].

A number of limitations should be noted when interpreting and generalizing these findings. First, we restricted our sample to very poor populations for which Demographic and Health Surveys have collected anthropometric and wealth data. This restricted our sample to populations living in tropical and subtropical regions, and potentially attenuated the effect of contemporary climate relative to genetic population structure [49]. However, the raw association of temperature observed in our sample had a magnitude comparable to associations observed in studies including a wider range of latitudes [1, 2]. This suggests that the sample restriction did not attenuate the effect of contemporary climate. Moreover, despite this restriction we still observed substantial variation in basal BMI—from approximately 17 to 23—which is comparable in magnitude to that observed in prior studies [1, 2]. This indicates that the restriction to a tropical and subtropical sample did not place an artificial ceiling on body mass indices. That said, the current findings only apply to those populations residing in tropical and subtropical regions. Another concern raised by the sample is the restriction to poor populations to estimate body form prior to the nutrition transition. It is well known that poverty-induced malnutrition is associated with stunting [50]. Thus, the basal BMI and weight-for-height is estimated on populations that have likely experienced less stature growth than well-nourished populations. This could lead to an overall upward bias in BMI and weight-for-height compared to well-nourished populations. However, given that the major outcome measures—bBMI and basal weight-for-height—are not strongly associated with wealth, it is unlikely that this upward bias could account for the current results. The Demographic and Health Surveys also have substantially more data from a wider range of countries for women than for men. Although, there is no clear explanation for this, we suspect that it is related to the heavy emphasis on maternal and child health in the surveys. Although this limits our ability to examine worldwide variation in men, there were sufficient surveys with male samples to validate the adult results across genders. Finally, it is possible that other unmeasured environmental variables that are arbitrarily correlated with genetic population structure can account for the observed relationship between genetic population structure and body form. In our analyses, we have attempted to rule out several such factors, including economic resources, urban residence, and mode of subsistence, and future work will hopefully examine other possible environmental confounders.

We show here that genetic population structure strongly predicts basal body form among both children and adults, which is consistent with past findings that a large portion of these population differences must arise early in development [10]. We also show that this is not associated with major subsistence type or current economic resources. It is possible that this population variation is due to other unmeasured ecological factors. However, these findings put strong constraints on what other potential environmental factors could be. Specifically, they would need both to covary strongly with genetic population structure and also to have an effect very early in development. In the absence of empirical evidence for alternative ecological explanations, the most parsimonious account for these population differences is likely genetic variation across populations which may have arisen as a result of selection in ancestral environments or from gene flow or random genetic drift.

## Supporting Information

S1 Fig Location of Samples. X-axis is longitude and Y-axis is latitude.
(DOCX)

S2 Fig Relationship between mean child basal WH and maximum temperature of hottest month and genetic affinity-predicted bWH (DOCX)

S1 Table Ethnolinguistic groups from DHS datasets matched to Tishkoff Ethnolinguistic groups. (DOCX)

S2 Table Sensitivity of Genetic-Affinity Models to Inclusion of Latitude and Longitude of groups. Including longitude and latitude in the genetic affinity model do not substantially modify the coefficients for the genetic affinity variables. * p < 0.05.
(DOCX)
