# Supplementary figures and images for: Genetic Population Structure Accounts for Contemporary Ecogeographic Patterns in Tropic and Subtropic-Dwelling Humans
Source: PLoS One. 2015 Mar 27;10(3):e0122301. doi: 10.1371/journal.pone.0122301 (PMC4376747; doi:10.1371/journal.pone.0122301)

**Figure S.1. Location of Samples. X-axis is longitude and Y-axis is latitude.**


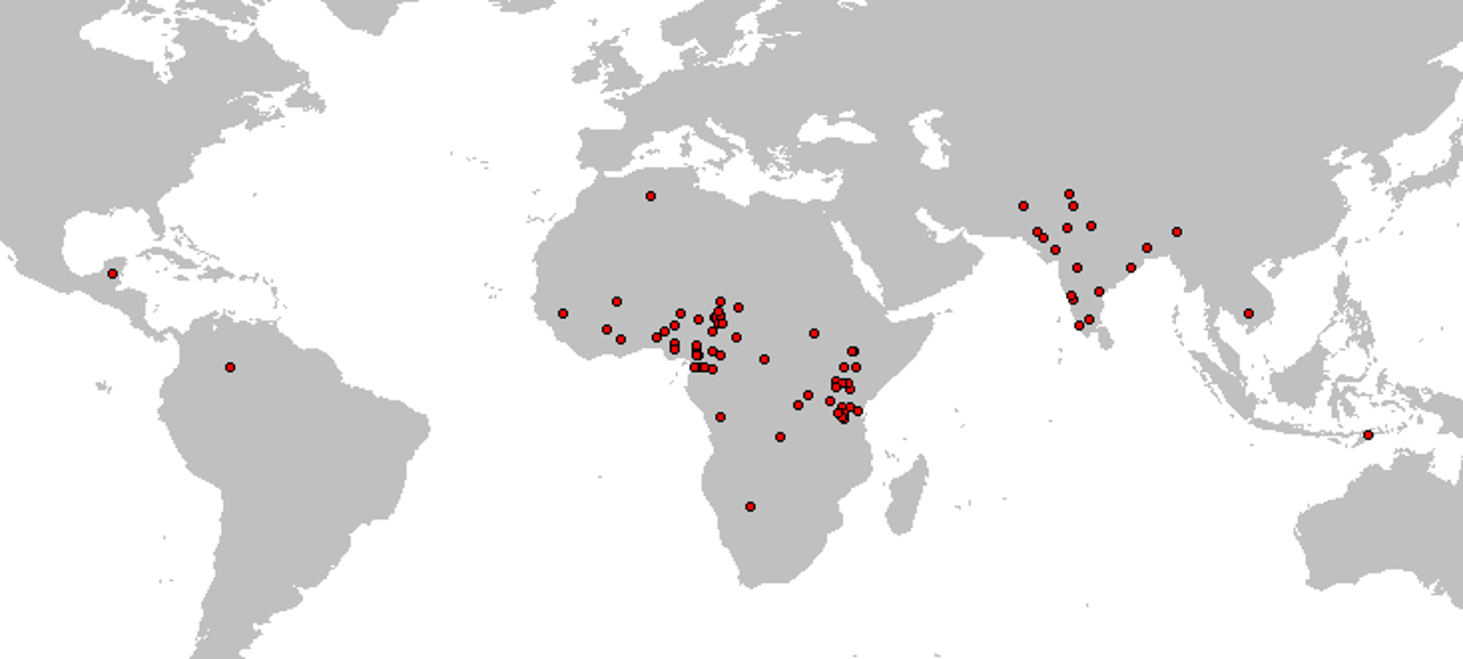

Supplement: S1 Fig — X-axis is longitude and Y-axis is latitude. (DOCX) [file pone.0122301.s001.docx]
