# Supplementary material for: Genetic Population Structure Accounts for Contemporary Ecogeographic Patterns in Tropic and Subtropic-Dwelling Humans
Source: PLoS One. 2015 Mar 27;10(3):e0122301. doi: 10.1371/journal.pone.0122301 (PMC4376747; doi:10.1371/journal.pone.0122301)
Supplement: S2 Fig — (DOCX) [file pone.0122301.s002.docx]

**Figure S.2. Relationship between mean child basal WH and maximum temperature of hottest month and genetic affinity-predicted bWH**

**
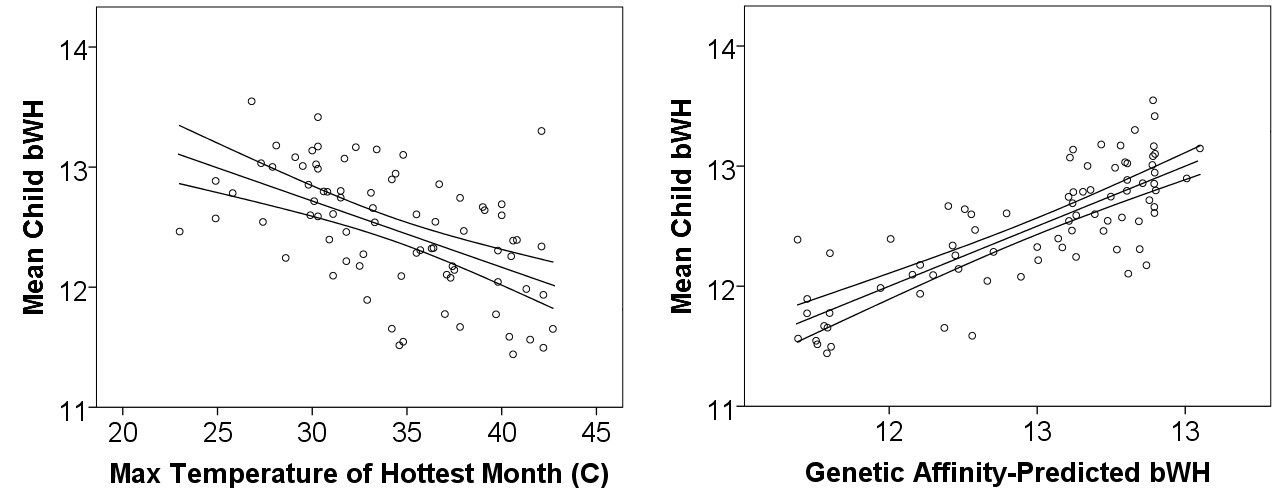
**
