# Supplementary material for: Genetic Population Structure Accounts for Contemporary Ecogeographic Patterns in Tropic and Subtropic-Dwelling Humans
Source: PLoS One. 2015 Mar 27;10(3):e0122301. doi: 10.1371/journal.pone.0122301 (PMC4376747; doi:10.1371/journal.pone.0122301)
Supplement: S1 Table — (DOCX) [file pone.0122301.s003.docx]

**Table S.1. Ethnolinguistic groups from DHS datasets matched to Tishkoff Ethnolinguistic groups**

| Ethnicity | |  |  | Temperature | | | Adult Female | | | Adult Male | | | Girl | | | Boy | | |
| --- | --- | --- | --- | --- | --- | --- | --- | --- | --- | --- | --- | --- | --- | --- | --- | --- | --- | --- |
| **Tishkoff** | **DHS** | **Lat** | **Long** | **Mean** | **Max** | **Min** | **bBMI** | **SE** | **N** | **bBMI** | **SE** | **N** | **bWH** | **SE** | **N** | **bWH** | **SE** | **N** |
| **Central Africa** | |  |  |  |  |  |  |  |  |  |  |  |  |  |  |  |  |  |
| Yakoma | Yakoma | 4.3 | 22.3 | 25.10 | 33.1 | 17.4 | 22.2 | 0.46 | 43 |  |  |  | 12.7 | 0.25 | 30 | 12.9 | 0.23 | 39 |
| Barega | Leg-Mwenga^1^ | -3.5 | 28 | 23.70 | 30.20 | 16.9 | 22.4 | 0.25 | 377 |  |  |  | 12.9 | 0.11 | 188 | 13.2 | 0.11 | 177 |
| Baluba | Kasai, Katanga^2^ | -9 | 25 | 22.60 | 31.10 | 13.3 | 21.7 | 0.29 | 333 |  |  |  | 12.4 | 0.12 | 170 | 12.9 | 0.11 | 184 |
| Kongo | Bakongo^1^ | -5.5 | 15 | 23.40 | 29.80 | 15.2 | 21.2 | 0.16 | 578 |  |  |  | 12.6 | 0.10 | 361 | 13.1 | 0.10 | 383 |
| Kanembou | Kanem-bornou | 14 | 15 | 28.20 | 40.40 | 13.6 | 19.3 | 0.17 | 410 |  |  |  | 11.5 | 0.11 | 284 | 11.7 | 0.11 | 267 |
| Bulala | Fitri-Batha^2^ | 13 | 18 | 29.40 | 42.20 | 14.8 | 19.7 | 0.22 | 212 |  |  |  | 11.8 | 0.13 | 152 | 12.1 | 0.14 | 132 |
| Baggara | Arab Choe^1^ | 12.5 | 14.5 | 27.50 | 40. | 13.5 | 19.8 | 0.61 | 22 |  |  |  | 12.2 | 0.36 | 11 | 13.0 | 0.32 | 17 |
| Massa | Massa | 10.3 | 15.3 | 28. | 39.10 | 17.5 | 21.5 | 0.46 | 42 |  |  |  | 12.4 | 0.25 | 32 | 12.9 | 0.24 | 34 |
| Sara_various | Sara | 8 | 17.5 | 26.60 | 37.80 | 14.5 | 21.6 | 0.12 | 981 |  |  |  | 12.6 | 0.08 | 705 | 12.9 | 0.08 | 681 |
| Mandara | Wandala/Mandara | 11.3 | 14 | 27. | 39. | 15.6 | 20.7 | 0.55 | 28 |  |  |  | 12.5 | 0.30 | 19 | 12.8 | 0.36 | 12 |
| Gbaya | Gbaya | 5 | 15 | 23.90 | 31.50 | 17.0 | 21.1 | 0.18 | 442 |  |  |  | 12.6 | 0.10 | 265 | 12.9 | 0.10 | 283 |
| Mbum | Mboum | 5.5 | 13.5 | 24. | 31.50 | 17.5 | 21.7 | 0.23 | 217 |  |  |  | 12.7 | 0.13 | 147 | 12.9 | 0.13 | 153 |
| Wimbum | Wimbum | 6.5 | 10.8 | 18.50 | 26.80 | 12.2 | 23.6 | 0.51 | 34 |  |  |  | 13.2 | 0.29 | 21 | 13.9 | 0.33 | 15 |
| Bamoun | Bamoun | 5.5 | 10.8 | 22.70 | 30.30 | 17.1 | 23.2 | 0.37 | 80 |  |  |  | 13.2 | 0.24 | 40 | 13.6 | 0.24 | 38 |
| Kanuri | Kanuri | 11.3 | 14.3 | 27.70 | 39.80 | 16.2 | 20.7 | 0.13 | 754 |  |  |  | 11.8 | 0.09 | 408 | 12.3 | 0.09 | 416 |
| Banen | Banen | 4.8 | 10.8 | 23.50 | 30.60 | 18.2 | 22.6 | 0.94 | 6 |  |  |  | 12.6 | 0.46 | 4 | 13.0 | 0.57 | 1 |
| Yambassa | Yambassa | 4.8 | 11.3 | 25.20 | 32.30 | 20.2 | 22.4 | 0.63 | 21 |  |  |  | 12.8 | 0.34 | 14 | 13.5 | 0.34 | 14 |
| Mvae | Beti^3^ | 3 | 12 | 23.50 | 29.50 | 18.3 | 22.3 | 0.33 | 121 |  |  |  | 12.8 | 0.19 | 71 | 13.2 | 0.20 | 74 |
| Bulu | Boulou-Fang^1^ | 3 | 11 | 23.50 | 29.10 | 18.3 | 22.7 | 0.52 | 35 |  |  |  | 12.8 | 0.32 | 18 | 13.3 | 0.30 | 20 |
| Ngumba | Meka^1^ | 3 | 10.3 | 25.10 | 30.10 | 20.5 | 22.5 | 0.43 | 48 |  |  |  | 12.3 | 0.24 | 35 | 13.1 | 0.27 | 27 |
| Baka | Pygmee^1^ | 2.5 | 13.5 | 23.20 | 30.30 | 17.3 | 21.7 | 0.70 | 15 |  |  |  | 12.6 | 0.39 | 8 | 12.6 | 0.39 | 9 |
| Nuer | Nuwer, Ethiopia | 8.5 | 31 | 27.50 | 37.50 | 18.6 | 18.4 | 0.28 | 213 | 18.5 | 0.33 | 64 | 12.0 | 0.13 | 129 | 12.3 | 0.13 | 141 |
| Tutsi/Hutu | Rwanda^2^ | -2 | 30 | 20.30 | 27.30 | 13.8 | 22.1 | 0.07 | 7813 | 21.2 | 0.18 | 2028 | 12.9 | 0.04 | 4116 | 13.2 | 0.04 | 4118 |
| Fulani_Cameroon | Peulh, Cameroon | 9 | 13.5 | 26.90 | 38. | 16.5 | 20.4 | 0.35 | 119 |  |  |  | 12.3 | 0.21 | 63 | 12.6 | 0.20 | 75 |
| Fulani_Mbororo | Peulh (Far North) | 11.8 | 14.8 | 27.80 | 40.50 | 14.8 | 19.4 | 0.50 | 36 |  |  |  | 12.0 | 0.28 | 23 | 12.5 | 0.32 | 17 |
| Hausa_Cameroon | Hausa, Cameroon | 10.5 | 14.5 | 28.40 | 40. | 18.7 | 21.4 | 0.69 | 20 |  |  |  | 12.3 | 0.40 | 8 | 13.0 | 0.37 | 12 |
| **Eastern Africa** | |  |  |  |  |  |  |  |  |  |  |  |  |  |  |  |  |  |
| Burji | Burji | 5.5 | 37.8 | 22.60 | 31.70 | 16.0 | 21.8 | 0.84 | 9 |  |  |  | 13.2 | 0.46 | 4 | 13.0 | 0.42 | 7 |
| Konso | Konso | 5.5 | 37.5 | 22.30 | 30.90 | 16.3 | 20.9 | 0.59 | 23 | 20.5 | 0.75 | 7 | 12.0 | 0.32 | 15 | 12.8 | 0.35 | 12 |
| Borana | Oromo^3^ | 3 | 38 | 26.30 | 36.30 | 17.7 | 20.0 | 0.08 | 3354 | 19.0 | 0.21 | 1156 | 12.1 | 0.06 | 1695 | 12.5 | 0.05 | 1788 |
| Maasai_Il'gwesi | Masai, Kenya | 0.3 | 36.8 | 17.40 | 27.40 | 7.4 | 20.2 | 0.29 | 117 |  |  |  | 12.4 | 0.17 | 81 | 12.7 | 0.17 | 76 |
| Nandi | Kalenjin^3^ | 0 | 35.5 | 16.50 | 25.80 | 8.6 | 21.0 | 0.12 | 1011 |  |  |  | 12.6 | 0.08 | 617 | 13.0 | 0.08 | 650 |
| Kikuyu | Kikuyu | -1 | 37 | 19. | 28.10 | 10.9 | 21.7 | 0.18 | 1111 |  |  |  | 13.0 | 0.12 | 589 | 13.3 | 0.12 | 567 |
| Luhya | Luhya | 0.5 | 34.5 | 21.80 | 30.30 | 14.4 | 21.8 | 0.13 | 1011 |  |  |  | 13.0 | 0.09 | 648 | 13.4 | 0.09 | 639 |
| Luo | Luo | -0.5 | 34.5 | 21.90 | 30.30 | 14.1 | 21.4 | 0.11 | 1304 |  |  |  | 12.9 | 0.07 | 710 | 13.1 | 0.07 | 770 |
| Turkana | Turkana | 3 | 36 | 28.70 | 36.40 | 21.3 | 19.6 | 0.43 | 49 |  |  |  | 12.2 | 0.26 | 27 | 12.4 | 0.24 | 35 |
| Gogo | Gogo | -6 | 36 | 22.50 | 31.80 | 12.5 | 21.3 | 0.40 | 59 |  |  |  | 12.2 | 0.22 | 43 | 12.8 | 0.20 | 55 |
| Iraqw | Iragw^4^ | -4 | 35.5 | 16. | 23. | 8.1 | 22.3 | 0.42 | 53 |  |  |  | 12.4 | 0.23 | 39 | 12.5 | 0.23 | 38 |
| Maasai | Maasai, Tanzania | -4 | 37 | 19.90 | 28.60 | 11.9 | 21.0 | 0.53 | 62 |  |  |  | 12.0 | 0.22 | 39 | 12.5 | 0.19 | 57 |
| Burunge | Mbulu^5^ | -5.3 | 36 | 21.40 | 30. | 11.6 | 21.8 | 0.83 | 9 |  |  |  | 12.8 | 0.48 | 3 | 13.5 | 0.44 | 6 |
| Pare | Pare | -4.5 | 38.3 | 16.30 | 24.90 | 9.1 | 21.3 | 0.46 | 46 |  |  |  | 12.8 | 0.25 | 34 | 12.9 | 0.26 | 31 |
| Rangi | Rangi | -5 | 36 | 21.40 | 29.90 | 11.8 | 21.0 | 0.61 | 22 |  |  |  | 12.4 | 0.35 | 12 | 12.8 | 0.33 | 16 |
| Sambaa | Sambaa | -4.5 | 38.3 | 16.30 | 24.90 | 9.1 | 21.5 | 0.43 | 49 |  |  |  | 12.6 | 0.23 | 38 | 12.6 | 0.26 | 29 |
| Sandawe | Sandawe | -5.5 | 35.5 | 23.20 | 31.80 | 13.4 | 20.7 | 0.93 | 6 |  |  |  | 12.0 | 0.44 | 5 | 12.4 | 0.48 | 4 |
| Sukuma | Sukuma | -3 | 33.5 | 22.90 | 30.80 | 15.1 | 21.8 | 0.21 | 261 |  |  |  | 12.7 | 0.12 | 187 | 12.9 | 0.12 | 192 |
| Turu | Turu/Nyaturu | -5 | 35 | 20.10 | 27.90 | 11.1 | 21.8 | 0.64 | 19 |  |  |  | 12.5 | 0.31 | 17 | 13.5 | 0.32 | 17 |
| **Saharan Africa** | |  |  |  |  |  |  |  |  |  |  |  |  |  |  |  |  |  |
| Mozabite | Morocco^2^ | 32 | 3 | 21.40 | 42.10 | 3.3 | 22.2 | 0.11 | 4490 |  |  |  | 13.1 | 0.09 | 1933 | 13.5 | 0.08 | 2167 |
| **Southern Africa** | |  |  |  |  |  |  |  |  |  |  |  |  |  |  |  |  |  |
| San | San | -21 | 20 | 20.60 | 32.50 | 4.1 | 20.0 | 0.45 | 45 |  |  |  | 12.0 | 0.16 | 106 | 12.3 | 0.16 | 96 |
| **Western Africa** | |  |  |  |  |  |  |  |  |  |  |  |  |  |  |  |  |  |
| Yoruba | Yoruba | 8 | 4 | 26.20 | 34.40 | 19.5 | 21.6 | 0.11 | 1872 |  |  |  | 12.8 | 0.08 | 1217 | 13.1 | 0.08 | 1198 |
| Dioula | Dioula | 9.5 | -4.5 | 27. | 35.70 | 18.3 | 21.3 | 0.08 | 6332 | 21.1 | 0.48 | 82 | 12.1 | 0.04 | 4075 | 12.5 | 0.04 | 4260 |
| Brong | Akan^3^ | 7.5 | -2 | 25.40 | 33.30 | 19.5 | 21.1 | 0.09 | 3037 |  |  |  | 12.3 | 0.06 | 24 | 12.8 | 0.06 | 27 |
| Dogon | Dogon | 14 | -3 | 28.30 | 39.80 | 15.6 | 21.8 | 0.12 | 9 |  |  |  | 12.1 | 0.08 | 588 | 12.5 | 0.08 | 610 |
| Igala | Igala | 7 | 7 | 27.40 | 34.80 | 21.1 | 21.9 | 0.36 | 127 |  |  |  | 12.9 | 0.24 | 52 | 13.3 | 0.23 | 64 |
| Mandinka | Mandigue^1^ | 12 | -12 | 25.80 | 36.50 | 15.2 | 21.5 | 0.24 | 253 | 21.1 | 0.40 | 121 | 12.3 | 0.14 | 223 | 12.7 | 0.13 | 258 |
| Gwari | Gwari | 10 | 7 | 26.50 | 36.70 | 17.0 | 21.3 | 0.37 | 134 |  |  |  | 12.7 | 0.22 | 67 | 13.0 | 0.24 | 51 |
| Igbo | Igbo | 6 | 7 | 26.80 | 33.20 | 21.8 | 20.9 | 0.19 | 487 |  |  |  | 12.4 | 0.10 | 477 | 12.9 | 0.10 | 509 |
| Bassange | Nupe^1^ | 9 | 5.5 | 28.10 | 37.40 | 19.3 | 21.0 | 0.43 | 113 |  |  |  | 11.9 | 0.25 | 48 | 12.4 | 0.24 | 51 |
| Fulani_Nigeria | Fulani, Nigeria | 11 | 11 | 25.30 | 37.30 | 12.2 | 20.1 | 0.12 | 1079 |  |  |  | 11.9 | 0.09 | 520 | 12.3 | 0.08 | 577 |
| Hausa_Nigeria | Hausa, Nigeria | 12 | 8 | 25.40 | 37.10 | 13.0 | 20.5 | 0.10 | 2889 |  |  |  | 12.0 | 0.07 | 2029 | 12.2 | 0.07 | 1933 |
| **Americas** | |  |  |  |  |  |  |  |  |  |  |  |  |  |  |  |  |  |
| Maya | Guatemala, Indian | 19 | -89 | 25.40 | 34.20 | 16.1 | 23.1 | 0.13 | 1533 |  |  |  | 12.7 | 0.08 | 1209 | 13.1 | 0.08 | 1240 |
| Colombian | Native Colombian | 3 | -69 | 26.50 | 33.40 | 21.3 | 24.4 | 0.15 | 749 |  |  |  | 13.0 | 0.10 | 397 | 13.3 | 0.10 | 419 |
| **Southeast Asia** | |  |  |  |  |  |  |  |  |  |  |  |  |  |  |  |  |  |
| Cambodian | Cambodia | 12 | 105 | 27.80 | 34.70 | 21.0 | 20.3 | 0.08 | 6389 |  |  |  | 11.9 | 0.05 | 2945 | 12.3 | 0.05 | 3060 |
| Melanesian | Timor L'Este^2^ | -8.84 | 125.6 | 24.50 | 31.10 | 17.9 | 19.7 | 0.10 | 2258 |  |  |  | 11.9 | 0.07 | 1265 | 12.3 | 0.07 | 1334 |
| **Southern Asia** | |  |  |  |  |  |  |  |  |  |  |  |  |  |  |  |  |  |
| Bengali | West Bengal, Bengali | 23.3 | 87.7 | 26.40 | 37.80 | 12.6 | 18.9 | 0.07 | 17849 | 19.0 | 0.20 | 1927 | 11.4 | 0.04 | 6846 | 11.9 | 0.04 | 7259 |
| Kannada | Kannada | 15 | 75 | 25.20 | 34.60 | 16.6 | 18.3 | 0.13 | 1750 | 18.7 | 0.26 | 1031 | 11.2 | 0.11 | 530 | 11.8 | 0.10 | 612 |
| Malayalam | Malayalam | 10 | 76.3 | 27.30 | 32.90 | 22.3 | 19.3 | 0.31 | 266 | 19.9 | 0.53 | 153 | 11.7 | 0.16 | 217 | 12.1 | 0.16 | 212 |
| Sindhi | Sindhi | 26 | 69 | 26.90 | 42.70 | 7.4 | 19.8 | 0.30 | 160 | 18.8 | 1.01 | 2 | 11.6 | 0.14 | 238 | 11.7 | 0.13 | 247 |
| Marathi | Marathi | 19.8 | 75.9 | 26.60 | 40.60 | 14.0 | 18.2 | 0.14 | 1494 | 18.4 | 0.27 | 995 | 11.1 | 0.11 | 512 | 11.7 | 0.11 | 598 |
| Oriya | Oriya | 20 | 85 | 26.10 | 34.80 | 14.8 | 18.3 | 0.09 | 2541 | 18.7 | 0.28 | 406 | 11.4 | 0.07 | 966 | 11.7 | 0.07 | 1110 |
| Punjabi | Punjabi | 30.5 | 75.3 | 24.40 | 41.30 | 5.3 | 19.8 | 0.25 | 1013 | 20.0 | 0.53 | 123 | 11.6 | 0.12 | 790 | 12.3 | 0.12 | 840 |
| Telugu | Telugu | 15.9 | 79.6 | 28.50 | 39.70 | 18.1 | 18.4 | 0.11 | 2761 | 18.8 | 0.24 | 1368 | 11.6 | 0.12 | 461 | 12.0 | 0.11 | 515 |
| Rajasthan | Rajasthan | 26.7 | 74.3 | 25.60 | 40.60 | 7.7 | 19.4 | 0.78 | 11 |  |  |  | 12.0 | 0.42 | 6 | 12.8 | 0.57 | 1 |
| Tamil | Tamil | 11.1 | 78 | 28.50 | 37. | 20.3 | 18.7 | 0.14 | 1872 | 19.1 | 0.28 | 1125 | 11.6 | 0.14 | 364 | 12.0 | 0.13 | 405 |
| Hindi | Hindi | 27 | 78.4 | 25.70 | 42.20 | 7.5 | 18.8 | 0.07 | 19812 | 18.7 | 0.17 | 5587 | 11.3 | 0.04 | 8898 | 11.7 | 0.04 | 9782 |
| Assamese | Assamese | 26 | 93 | 24.40 | 32.70 | 10.0 | 19.1 | 0.17 | 671 | 18.8 | 0.44 | 169 | 11.9 | 0.12 | 276 | 12.6 | 0.12 | 292 |
| Konkani | Konkani | 14.6 | 75.1 | 24.90 | 34.20 | 16.2 | 17.6 | 0.26 | 307 | 19.0 | 0.52 | 142 | 11.5 | 0.17 | 191 | 11.8 | 0.17 | 183 |
| Gujarat | Gujarat | 23 | 72 | 27.40 | 41.50 | 11.0 | 17.9 | 0.16 | 1377 | 18.4 | 0.39 | 273 | 11.3 | 0.10 | 658 | 11.8 | 0.11 | 693 |
| Kashmiri | Kashmiri | 32.4 | 74.5 | 23.70 | 40.80 | 5.5 | 19.7 | 0.30 | 788 | 19.4 | 0.53 | 138 | 12.3 | 0.18 | 203 | 12.5 | 0.18 | 239 |
| Pathan | Pathan | 25 | 70 | 26.70 | 42.10 | 7.5 | 22.7 | 0.34 | 243 |  |  |  | 12.2 | 0.15 | 334 | 12.4 | 0.15 | 354 |
| Balochi | Balochi | 30.5 | 66.5 | 17.20 | 35.50 | -3.8 | 20.6 | 0.44 | 57 |  |  |  | 12.1 | 0.23 | 45 | 12.4 | 0.22 | 50 |
| Brahui | Brahui | 30.5 | 66.5 | 17.20 | 35.50 | -3.8 | 23.4 | 0.39 | 62 |  |  |  | 12.3 | 0.20 | 71 | 12.9 | 0.22 | 51 |

1. Names for the same ethnic group or language.
2. Ethnic group defined by location
3. Subgroup
4. Misspelling in DHS
5. Closest linguistic group
