# Supplementary material for: Genetic Population Structure Accounts for Contemporary Ecogeographic Patterns in Tropic and Subtropic-Dwelling Humans
Source: PLoS One. 2015 Mar 27;10(3):e0122301. doi: 10.1371/journal.pone.0122301 (PMC4376747; doi:10.1371/journal.pone.0122301)
Supplement: S2 Table — Including longitude and latitude in the genetic affinity model do not substantially modify the coefficients for the genetic affinity variables. * p < 0.05. (DOCX) [file pone.0122301.s004.docx]

**Table S.2. Sensitivity of Genetic-Affinity Models to Inclusion of Latitude and Longitude of groups.**  Including longitude and latitude in the genetic affinity model do not substantially modify the coefficients for the genetic affinity variables. * p < 0.05.

|  | **Female bBMI** | | **Boy bWH** | | **Girl bWH** | |
| --- | --- | --- | --- | --- | --- | --- |
|  | **Genetic Affinity** | **Genetic Affinity**  **+Lat/Long** | **Genetic Affinity** | **Genetic Affinity**  **+Lat/Long** | **Genetic Affinity** | **Genetic Affinity**  **+Lat/Long** |
| **Intercept** | 22.2 (21.8,22.5) | 21.9 (21.4,22.3) | 13.2 (13.0,13.3) | 12.6 (12.4,12.8) | 12.8 (12.6,12.9) | 12.6 (12.4,12.8) |
| **Fulani** | -4.5 (-6.5,-2.5)* | -4.5 (-6.5,-2.5)* | -1.0 (-2.0,-0.1)* | -1.1 (-2.0,-0.2)* | -1.1 (-2.0,-0.2)* | -1.1 (-2.0,-0.2)* |
| **Nilo-Saharan** | -4.3 (-6.5,-2.2)* | -5.6 (-7.8,-3.3)* | -0.5 (-1.6,0.5) | -0.8 (-1.9,0.2) | -0.4 (-1.3,0.6) | -0.8 (-1.9,0.2) |
| **Oceania** | -2.9 (-5.5,-0.2)* | -6.1 (-9.5,-2.7)* | -1.0 (-2.2,0.3) | -2.1 (-3.7,-0.6)* | -0.9 (-2.1,0.3) | -2.1 (-3.7,-0.6)* |
| **Chadic** | -4.3 (-6.4,-2.2)* | -4.4 (-6.6,-2.2)* | -1.9 (-2.8,-0.9)* | -1.9 (-2.9,-0.9)* | -1.8 (-2.7,-0.9)* | -1.9 (-2.9,-0.9)* |
| **S. African Khoesan** | -2.7 (-4.5,-0.8)* | -2.5 (-4.7,-0.3)* | -1.0 (-1.9,-0.6)* | -0.8 (-1.8,0.2) | -0.9 (-1.8,-0.1)* | -0.8 (-1.8,0.2) |
| **East Asian** | -1.8 (-3.7,0.01)* | -4.8 (-7.7,-1.8)* | -0.8 (-1.7,0.1) | -2.1 (-3.4,-0.8)* | -0.8 (-1.7,0.0) | -2.1 (-3.4,-0.8)* |
| **Sandawe** | -1.4 (-3.5,0.6) | -2.5 (-4.7,-0.4)* | -0.4 (-1.4,0.6) | -1.1 (-2.1,-0.1)* | -0.7 (-1.6,-1.1)* | -1.1 (-2.1,-0.1)* |
| **Indian** | -4.1 (-4.7,-3.5)* | -6.4 (-8.4,-4.4)* | -1.3 (-1.6,-1.0)* | -2.4 (-3.3,-1.5)* | -1.4 (-1.7,-1.1)* | -2.4 (-3.3,-1.5)* |
| **Cushitic** | -0.9 (-1.9,0.1) | -1.7 (-2.8,-0.6)* | -0.4 (-0.9,0.1) | -0.6 (-1.1,-0.1)* | -0.2 (-0.7,0.2) | -0.6 (-1.1,-0.1)* |
| **W. Pygmy** | -0.4 (-2.8,2.0) | -0.5 (-2.8,1.8) | -0.8 (-1.9,0.4) | -0.2 (-1.3,0.8) | -0.2 (-1.3,0.9) | -0.2 (-1.3,0.8) |
| **European** | 2.8 (1.3,4.3)* | 2.3 (-0.1,4.7) | 0.1 (-0.6,0.9) | -0.0 (-1.1,1.1) | 0.3 (-0.4,1.0) | -0.0 (-1.1,1.1) |
| **N. American** | 2.6 (1.0,4.1)* | 6.6 (3.4,9.8)* | 0.3 (-0.5,1.0) | 2.0 (0.63.5)* | 0.4 (-0.3,1.0) | 2.0 (0.5,3.5)* |
| **Latitude** |  | 0.01 (-0.03,0.05) |  | 0.01 (-0.01,0.03) |  | 0.01 (-0.01,0.02) |
| **Longitude** |  | 0.03 (0.01,0.05)* |  | 0.01 (0.00,0.02)* |  | 0.01 (0.00,0.02)* |
